# Hygiene inspections on passenger ships in Europe - an overview

**DOI:** 10.1186/1471-2458-10-122

**Published:** 2010-03-10

**Authors:** Varvara A Mouchtouri, Sandra Westacott, Gordon Nichols, Tobias Riemer, Mel Skipp, Christopher LR Bartlett, Jenny Kremastinou, Christos Hadjichristodoulou, the SHIPSAN partnership**

**Affiliations:** 1 Department of Hygiene and Epidemiology, Faculty of Medicine, University of Thessaly, Larissa, Greece; 2 Association of Port Health Authorities, Southampton Port Health Authority, London, UK; 3 Gastrointestinal, Emerging and Zoonotic Infections Department Health Protection Agency, Centre for Infections, London, UK; 4 Hamburg Port Health Center, Institute of Occupational and Maritime Medicine, Hamburg, Germany; 5 European Cruise Council and Cruise Line International Association; 6 University College London, Centre for Infectious Disease Epidemiology Department of Primary Care and Population Sciences Royal Free and University College Medical School, London, UK; 7 Department of Public and Administrative Health, National School of Public Health, Athens, Greece

## Abstract

**Background:**

Hygiene inspections on passenger ships are important for the prevention of communicable diseases. The European Union (EU) countries conduct hygiene inspections on passenger ships in order to ensure that appropriate measures have been taken to eliminate potential sources of contamination which could lead to the spread of communicable diseases. This study was implemented within the framework of the EU SHIPSAN project and it investigates the legislation applied and practices of hygiene inspections of passenger ships in the EU Member States (MS) and European Free Trade Association countries.

**Methods:**

Two questionnaires were composed and disseminated to 28 countries. A total of 92 questionnaires were completed by competent authorities responsible for hygiene inspections (n = 48) and the creation of legislation (n = 44); response rates were 96%, and 75.9%, respectively.

**Results:**

Out of the 48 responding authorities responsible for hygiene inspections, a routine programme was used by 19 (39.6%) of these to conduct inspections of ships on national voyages and by 26 (54.2%) for ships on international voyages. Standardised inspection forms are used by 59.1% of the authorities. A scoring inspection system is applied by five (11.6%) of the 43 responding authorities. Environmental sampling is conducted by 84.1% of the authorities (37 out of 44). The inspection results are collected and analysed by 54.5% (24 out of 44) of the authorities, while 9 authorities (20.5%) declared that they publish the results. Inspections are conducted during outbreak investigations by 75% and 70.8% of the authorities, on ships on national and international voyages, respectively. A total of 31 (64.6%) and 39 (81.3%) authorities conducted inspections during complaint investigations on ships on international and on national voyages, respectively. Port-to-port communication between the national port authorities was reported by 35.4% (17 out of 48) of the responding authorities and 20.8% (10 out of 48) of the port authorities of other countries.

**Conclusion:**

This study revealed a diversity of approaches and practices in the conduct of inspections, differences in the qualifications/knowledge/experience of inspectors, the legislation applied during inspections, and the lack of communication and training among many EU countries. An integrated European inspection programme involving competent expert inspectors in each EU Member States and special training for ship hygiene delivered to crew members and inspectors would help to minimize the risk of communicable diseases. Common inspection tools at a European level for hygiene inspection practices and port-to-port communication are needed.

## Background

Inspections are applied in various fields of human activity, where standards and rules have been set, such as health and safety, engineering and environmental health. An inspection is a procedure conducted in order to examine establishments, processes, products, systems and records. Overall, it aims at assessing conditions and operations in relation to specific standards. Inspections can be conducted by governmental agencies or private companies as an external examination or audit. Additionally, inspections might be conducted internally by those who apply the standards, a practice known as "self auditing".

As part of national environmental health systems, hygiene inspections are conducted by competent governmental agencies in order to verify compliance with legal requirements, usually focusing on food businesses and food safety [1]. However, hygiene in establishments such as passenger ships extends beyond food safety, since other areas of public health importance are also present. These include, amongst others, potable and recreational water, waste management, legionnaires' disease prevention, pest management and the condition of accommodation spaces. Consequently, a hygiene inspection aboard a ship is a complex procedure, involving many of the aforementioned aspects and requiring comprehensive knowledge on the part of the inspector.

In the past, foodborne outbreaks on board passenger ships have been linked to inadequate food temperature control, infected food handlers, contaminated raw ingredients, cross contamination and inadequate heat treatment of food [2]. Risk factors for waterborne outbreaks have involved contaminated port water, inadequate treatment, improper loading techniques, poor design and maintenance of storage tanks, ingress of contamination during repair and maintenance, cross-connections, back siphonage, and insufficient residual disinfectant [3].

Other scientific studies have examined environmental health issues on board ships and have shown the colonization of water distribution systems with *Legionella *spp. on ferries [4,5], faecal bacteria in the potable water supply on merchant ships [6], contaminated ice and swimming pool water on cruise ships [7] and pest infestations on merchant ships including ferries [8,9].

Hygiene inspections are therefore necessary in order to ensure that satisfactory hygiene practices are applied properly and to ensure that appropriate measures have been taken to control public health risks that could lead to the introduction, transmission or spread of communicable diseases on ships. Many passenger ship companies have developed their own hygiene systems and implement rigorous audit inspections in order to assess hygiene conditions and to reduce the risk of communicable diseases.

EU countries conduct hygiene inspections according to their national legislation. Following the issue of the International Health Regulations (IHR) 2005 [10], some European countries such as France http://www.sante.gouv.fr/htm/dossiers/reglement_sanit_intern/accueil.htm, the Netherlands http://www.shipsanitation.nl/, Germany and the United Kingdom http://www.apha.org.uk have started developing existing programmes for ship inspections and have adjusted their national legislation in order to incorporate these Regulations. The scope of the IHR 2005 is to prevent, protect against, control and provide a public health response to the international spread of disease. According to IHR 2005, competent authorities conduct ship inspections in order to identify shipborne public health risks. Findings and control measures are recorded on the Ship Sanitation Control Exemption Certificate/Ship Sanitation Control Certificate, which carries a six month period of validity [11].

In 2002, the European Parliament and the Council adopted a Decision establishing a programme of European Community action in the field of public health 2003-2008 http://ec.europa.eu/health/ph_programme/programme_en.htm. One of the general objectives of this programme was to enhance the capability of responding rapidly and in a coordinated fashion to health related threats. Since passenger ships move continuously, visiting ports in different countries and continents, the need for coordinated action within the European Union is clear, especially if infections or other public health risks occur aboard passenger ships. Eurostat data show that there were about 410 million passenger visits through European Union (EU) ports in 2007 [12]. In 2006, the European project SHIPSAN was funded by the Directorate General for Health and Consumers of the European Commission in order to address such ship related health issues.

One of the tasks of the EU SHIPSAN project study is assessing the usefulness of an integrated common programme for communicable disease surveillance and hygiene inspections in Europe http://www.shipsan.eu. The assessment methodology included the description of the current situation regarding the inspection practices of the authorities and the relevant legislation. In addition, it included the identification of capacities, gaps and needs in hygiene inspections of passenger ships among the EU countries (EU Member States and European Free Trade Association-EFTA-countries).

This paper presents the results of a study conducted on competent authorities of 30 countries responsible for conducting hygiene inspections and creating legislation. It discusses the findings of this study and outlines the EU SHIPSAN project partnership proposals. The proposals concern the development of a programme with consistent approach to hygiene inspections on passenger ships in the EU, in order to prevent communicable diseases on passenger ships (such as foodborne and waterborne infections).

## Methods

### Data Collection

The data collection process was implemented in two phases and lasted from August 2007 until February 2008. During the first phase, competent authorities responsible for creating legislation and conducting hygiene inspections were identified in order to provide accurate information for the study purposes. A questionnaire was disseminated to the Ministry of Health or national surveillance centre of each country. Contact details for 50 national and regional competent authorities were identified in 30 countries (Austria, Belgium, Bulgaria, Croatia, Cyprus, Czech Republic, Denmark, Estonia, Finland, France, Germany, Greece, Hungary, Iceland, Ireland, Italy, Latvia, Lithuania, Malta, Norway, Poland, Portugal, Romania, Slovakia, Slovenia, Spain, Sweden, the Netherlands, Turkey and United Kingdom).

In the second phase of the data collection process, two questionnaires were constructed, pilot tested and disseminated to the competent authorities, which were identified during the first phase, in order to collect data on legislation and hygiene inspection practices.

In the first questionnaire, competent authorities were asked to provide information on the legislation they enforce: i) specific legislation, regulations or guidelines for cruise ships and ferries, ii) national legislation, regulations or guidelines for land-based premises including specific provisions for cruise ships and ferries, iii) national legislation, regulations or guidelines for land based premises also applicable to cruise ships and ferries and iv) other types of legislation.

The questionnaire for hygiene inspection practices asked for information on: i) responsibilities including 10 issues (food safety, potable water safety, waste management, pest control, accommodation spaces, housekeeping, medical facilities, recreational water safety, air handling and ventilation and other issue) that the competent authorities have the power to inspect, ii) frequency of inspections, iii) port-to-port communication, iv) environmental sampling, v) inspection tools used such as standardised forms and equipment, vi) training needs, vii) gaps on hygiene inspections, and viii) statistical data (Additional file 1). No ethical approval or permission was required for this study to enable the collection and analysis of the data.

### Data analysis

The data collected were entered into a specifically designed database using EPI Info Version 3.01 and descriptive and correlation analyses were conducted. The chi-square or the Fischer exact tests were used to compare qualitative variables. Results were considered statistically significant when the P value was <0.05.

EU countries were divided into four priority groups (Group A, B, C and D) depending on the number of passenger ships sailing in their country, volume of passenger visits and number of ports. The following countries were included in Group A (having higher numbers of passenger movements, ships, ports than groups B, C and D): Denmark, France, Germany, Greece, Italy, Spain, Sweden and the United Kingdom. Group B (having higher numbers of passenger movements, ships, ports than groups C and D) included Belgium, Croatia, Estonia, Finland, Ireland, the Netherlands, Norway, Poland and Portugal. Group C (having higher numbers of passenger movements, ships, ports than group D) consisted of Bulgaria, Cyprus, Latvia, Lithuania, Malta, Iceland, Romania and Slovenia. The countries of Group D (having lower numbers of passenger movements, ships, ports than groups A, B and C) were Austria, Czech Republic, Hungary and Slovakia.

Additionally, EU countries were categorised as old or new Member States. The old ones consist of Austria, Belgium, Denmark, Finland, France, Germany, Greece, Iceland, Ireland, Italy, Norway, Portugal, the Netherlands, Spain, Sweden and United Kingdom. The new ones consist of Bulgaria, Cyprus, Czech Republic, Estonia, Hungary, Latvia, Lithuania, Malta, Poland, Romania, Slovakia, Slovenia and Croatia. The data were analyzed according to the groups A, B, C and D and old and new EU Member States.

The Department of Hygiene and Epidemiology of the University of Thessaly in Greece was responsible for the collection, analysis and presentation of the data. All other partners of the EU SHIPSAN project, which are presented in the acknowledgement section of this paper, were responsible for collecting data, reviewing result reports and developing proposals.

## Results

There were 44 completed questionnaires collected out of the total of 58 questionnaires related to hygiene legislation applied during inspections of cruise ships and ferries (response rate 75.9%) and these were from 28 countries. In this study, there are no data on legislation enforced by competent authorities in Belgium and Turkey.

A total of 48 completed questionnaires for hygiene inspection practices were collected out of the total of 50 questionnaires which were disseminated (response rate 96%) and these related to 28 countries. This study does not include data on inspection practices for Belgium and Romania.

Not all questions were answered by the 48 competent authorities responsible for hygiene inspections and by the 44 competent authorities responsible for the creation of legislation. Thus, the percentages were calculated using the number of authorities that answered the specific question as the denominator (excluding the missing values).

### Competent authorities in the EU

The results of this study revealed that in the EU Member States and the EFTA countries, the type of competent authorities responsible for conducting ship hygiene inspections differed from country to country. In particular the competent authorities included: a) departments within regional health authorities, b) local authority departments, c) ministries of health, d) ministries of the environment, e) ministries of trade and maritime affairs, f) private companies assigned by health departments and g) national food safety authorities. In some ports, authorities responsible for occupational health, communicable disease surveillance and environmental health have been merged into one authority. In other ports, committees with representatives from different authorities have been established and conducted combined inspections.

### Legislation and power to inspect

The majority of the authorities have the power to enforce national legislation, which is relevant to land based establishments, to ships on national (77.1%, 37 out of 48 of the authorities) and international voyages (56.3%, 27 out of 48 of the authorities). Specific national legislation for ships is enforced in 12 out of the 48 (25%) authorities for ships on national voyages and in 14 out of 48 authorities (29.2%) for ships on international voyages. About 27% (13 out of 48) of the authorities have reported that they have no legislation to enforce for ships on international voyages (Table 1).

**Table 1:** Legislation enforced by authorities on passenger ships according to the itinerary (national or international)

Legislation that the authorities (n = 48) have the power to enforce	National voyage		International voyage	
	
	Yes	%	Yes	%
Specific national legislation for ships	12	25	14	29.2
National legislation for land based establishments	37	77.1	27	56.3
No legislation	2	4.2	13	27.1
Other	4	8.3	5	10.4

The legislation enforced by competent authorities for food safety, potable and recreational water safety, waste management and pest control is presented in Table 2. The number of authorities that applied specific legislation for passenger ships for the six aforementioned topics ranged from two to six. There is no legislation for obtaining a food premises permit in 22.9% (11 out of 48) of the authorities. Greece and Italy applied specific legislation for food premise permits on ships. Cyprus requires ships to register food premises with the local authorities (Table 2).

**Table 2:** Legislation on hygiene issues which competent authorities of the EU and EFTA countries enforce according to its applicability to passenger ships

Topic	National legislation							
	
	specific for passenger ships		with provisions for passenger ships		without provisions for passenger ships		other	
	
	Yes/Total	%	Yes/Total	%	Yes/Total	%	Yes/Total	%
Food safety	3/45	6.7	4/45	8.9	31/45	68.9	7/45	15.6
Potable water safety	6/43	14.0	5/43	11.6	27/43	62.8	5/43	11.6
Recreational water safety	2/41	4.9	4/41	9.8	30/41	73.2	5/41	12.2
Waste management	6/38	15.8	2/38	5.3	22/38	57.9	8/38	21.1
Pest control	4/44	9.1	4/44	9.1	29/44	65.9	7/44	15.9
Clinical waste	4/39	10.3	4/39	10.3	25/39	64.1	6/39	15.4

### Responsibilities in hygiene inspections

Food safety and potable water safety aspects are inspected by 79.2% (38 out of 48) of the responding authorities, whereas pest control and waste management are inspected by 75% (36 out of 48) of the authorities. Medical facilities, air handling and ventilation are inspected by 60.4% (29 out of 48) of the authorities. Recreational water safety is inspected by 54.2% (26 out of 48) of the authorities and accommodation spaces and housekeeping aspects are inspected by 64.6% of the authorities (Table 3).

**Table 3:** Hygiene issues which authorities have the power to inspect on passenger ships

Hygiene issues that the authorities (n = 48) have the power to inspect	Yes	%
Food safety	38	79.2
Potable water safety	38	79.2
Waste management	36	75.0
Pest control	36	75.0
Accommodation spaces	31	64.6
Housekeeping	31	64.6
Medical facilities	29	60.4
Air handling and ventilation	29	60.4
Recreational water safety	26	54.2
Other*	11	22.9

*ballast water, premises for sport and playing, laundries, hairdressing salons and Legionnaires' disease prevention

Out of the 46 responding authorities, 12 have the power to inspect 9 items and 10 authorities 8 items. The rest of the 24 authorities (52.2%) have the power to inspect a range of two to five items from the 10 total which are included in the questionnaire (Table 3).

### Reasons for inspections

Out of the 48 responding authorities, 19 (39.6%) conduct inspections for ships on national voyages according to a routine programme and 26 (54.2%) for ships on international voyages (Table 4). Inspections are conducted during outbreak investigation by 75% and 70.8% of the authorities, on ships on national and international voyages, respectively. A total of 31 (64.6%) and 39 (81.3%) authorities conduct inspections during complaint investigation on ships on international and national voyages, respectively (Table 4). According to the comments noted in the questionnaires, some of the authorities considered the issuing of the certificates under the International Health Regulations (IHR) as a routine inspection programme (Table 4).

**Table 4:** Reasons why competent authorities (n = 48) conduct hygiene inspections on passenger ships

Inspection frequency	National voyage		International voyage	
	
	Yes	%	Yes	%
Outbreak investigation	36	**75.0%**	34	**70.8%**
Complaints investigation	31	**64.6%**	39	**81.3%**

Certificate issuing	28	**58.3%**	28	**58.3%**
Routine programme	19	**39.6%**	26	**54.2%**
Other*	2	**4.2%**	5	**10.4%**

* every year before the summer season, before Christmas, before Easter holidays

Seven (20%) of the 35 responding authorities did not provide any number of inspections during 2006, 12 (34.2%) authorities conducted from one to 50 passenger ship inspections, seven (20%) authorities from 51 to 100 inspections, five authorities from 101 to 240 inspections, two authorities from 241 to 700 inspections and two authorities from 701 to 2150 inspections.

### Hygiene inspection practices

Twenty three out of 43 (53%) responding authorities reported that they had specific critical issues in order to prevent a ship from sailing (Table 5). Standardised inspection forms are used by 59.1% of the authorities. A scoring inspection system is applied by five (11.6%) of the 43 responding authorities. Environmental sampling on passenger ships is conducted by 84.1% of the authorities (37 out of 44). The frequency of environmental sampling is presented in Table 5. The inspection results are collected and analysed by 54.5% (24 out of 44) of the authorities, while 9 authorities (20.5%) declared that they publish the results (Table 5).

**Table 5:** Inspection practices among EU countries

Subject area		Yes/Total	%
**Collection and analysis of inspection results**		24/44	54.5
**Inspection results publication**		9/44	20.5
**Critical enough issues to issue an order that a ship does not sail**		23/43	53.5
**On board measurements**		24/44	54.5
**Use of standardised inspection forms**		26/44	59.1
**Hygiene inspection scoring system**		5/43	11.6

**Environmental sampling**		37/44	84.1
**Frequency of environmental sampling:**	according to specific routine programme	11/37	29.7
	during outbreak investigation	29/37	78.4
	other*	19/37	51.4

**Port-to-port communication:**	among national port authorities	17/48	35.4
	among port authorities of other countries	10/48	20.8
	other**	14/48	29.2

*before issuing a certificate, yearly before the beginning of the summer

** in some countries communication with other country's port is through the Ministries of Health

### Port-to-port communication

Port-to-port communication among the national port authorities was reported by 35.4% (17 out of 48) of the responding authorities and 20.8% (10 out of 48) reported communication among port authorities of other countries (Table 5).

### Training

The personnel of 17 authorities (39.5%) have received training on hygiene inspections, while 11.1% has received training specifically for ship inspections. The majority of the responding authorities (73.2%) believe that specific training for passenger ship inspections is needed.

### Gaps reported

Twenty three of 44 (52.3%) authorities stated that the number of inspections conducted is insufficient and that there are gaps in the inspection systems (under-inspections, inconsistency of inspections, lack of coordination and overlapping, lack of personnel, inspections focus mainly on food safety and water safety). Gaps in legislation were reported by 11 out of 30 (36.7%) authorities.

### Comparison among the four categories grouped countries and old and new EU Member States

Inspection practices, which statistically significantly differed among the four categories of grouped countries, between old and new EU Member States and between regional and national authorities are presented in Table 6.

**Table 6:** Inspection practices which had statistical significant difference among the four categories of grouped countries and old and new EU Member States

		p-value		
		
Subject area	Question	Priority groups	Old versus new Member States	Regional versus National Authorities
**Hygiene issues that the authorities have the power to inspect**	Food safety	0.049		
	Accommodation spaces		0.01	
	Housekeeping		0.032	
	Air handling and ventilation		0.043	
**Legislation applied during inspections**	National legislation or regulations or guidelines for land based premises also applicable to cruise ships and ferries	0.051		
**Legislation related to obtaining a permit for food premises**	National legislation or regulations or guidelines for land based premises also applicable to cruise ships and ferries		0.059	
**Laws that the authorities have the power to apply on an international voyage**	National legislation for land based establishments			0.051
**Port-to-port communication**	No port-to-port communication among the national port authorities	0.037		0.001
**Standardised inspection forms**	Standardised inspection forms (checklists) are used during the inspections	0.012		
**Collection and analysis of inspection results**	Inspection results are collected and centrally analysed	0.011	0.02	
**Critical enough issues to issue an order that a ship does not sail**	There are sanitation issues that are considered critical enough to issue an order that a ship does not sail			0.05
**Gaps in hygiene inspections**	Reported gaps on hygiene issues	0.04		

Statistical significant differences were found among the categories of grouped countries regarding inspection powers (food safety, accommodation areas, housekeeping and air handling and ventilation), legislation applied during inspections, port-to-port communication, the use of a standardised inspection form and gaps reported on inspections (Table 6).

Of the 17 authorities that have received general training on hygiene inspections, six belong to priority group A, four belong to group B, five to group C and two to group D. Thirteen out of 23 authorities reporting gaps in inspection systems belonged to the old EU Member States group, whereas the other 10 belong to the new Member States group. Eight out of 12 (66.7%) responding authorities in Group A, 8 out of 14 (57.1%) in Group B and 7 out of 11 (63.6%) in group C believe that there are gaps in hygiene inspections, whereas the lower priority group D does not believe that there are any gaps. Ten out of the 23 authorities which believed that there are gaps in sanitation inspections are national authorities, while the remaining 13 were regional authorities.

## Discussion

The study results have revealed that there are diverse approaches and practices related to inspection as well as the legislation applied during inspections among the EU countries. Some countries have long established and well-developed national infrastructures for conducting inspections, while other countries have limited specific legislation for ships, since there is no significant passenger ship traffic in their ports.

The study results showed that many different authorities are responsible for conducting inspections within the same country without always having clearly defined roles and responsibilities. Simplified, less bureaucratic procedures and communications, as well as clearly defined roles and responsibilities can help to improve public health systems.

In our study, we explored the legislation standards applied during inspection. Several authorities were uncertain as to whether national legislation applies to ships on international voyages and declared that they have no legislation to enforce standards for ships on international voyages. In addition, several authorities were unclear as to whether the EU legislation applies to ships. There are some aspects such as potable water safety, where different risks exist on ships than on land due to particular conditions including bunkering, complicated piping systems, different sources of water in different ports or water production on board [3]. It is very important that inspection standards are based on legislation, otherwise inspected businesses will have opportunities to dispute the inspection results and if necessary appeal against enforcement decisions.

Inconsistent standards in legislation among different countries can create problems for the industry. For example, food temperature requirements may differ in the ports of call of a ship which creates confusion for all parties involved. As far as construction issues are concerned, it could be considered that as a ship sails all over the world, common globally accepted standards should exist. It is difficult, if not impossible, for a ship to change construction aspects depending on the port of call.

Our study results have revealed that a number of authorities have been given limited responsibilities to inspect specific aspects on board ships. Therefore, not all hygiene criteria are inspected and it is difficult to assess the level of compliance for the same ship for all different hygiene aspects on board. In addition, inspections are mainly focused on food and potable water safety, waste and pest management.

The Centers for Diseases Control and Prevention Vessel Sanitation Programme (CDC VSP) is an integrated standardised programme in the USA that has operated since 1975 and combines surveillance and standardised hygiene inspections. Other similar programmes were subsequently established in Canada http://www.hc-sc.gc.ca/hl-vs/travel-voyage/general/ship-navire-eng.php and Sydney [13,14]. According to publications, sanitation standards on cruise ships have been improved [15] and foodborne outbreaks declined [16] after implementation of the CDC VSP. The Port of Sydney programme has improved preventive action, and risk communication and management by cruise ship operators, and led to more timely investigation and support by public health authorities [13].

The frequency of inspections varies among the EU countries. Many authorities considered the issuance of the International Health Regulations 2005 Ship Sanitation Control Exemption Certificate/Ship Sanitation Control Certificate [10] as a routine inspection programme. Interim technical advice for inspection and issuance of ship sanitation certificates has been produced by the World Health Organization http://www.who.int/ihr/travel/TechnAdvSSC.pdf. This guidance is addressed to all types of ships, is based on International Labour Organization and International Maritime Organization conventions and WHO guidelines and provides questions and examples of evidence of conditions that can be found on board ships [11]. However, the EU legislation requires additional standards and therefore, inspections in the EU must ensure conformity with the EU legislation standards.

About half of the competent authorities collect and analyze the results from inspections. Useful information can be obtained and conclusions can be drawn by such analysis. For example, the monitoring of inspection results for each ship can help to prioritise the frequency of inspections. Adverse inspection results can be associated with outbreaks in the same establishment, inspectors' performance can be compared, risk factors can be identified [15], the implemented inspection system can be evaluated and problematic areas can be identified in order to focus on training. Central collection and analysis of data could help in decision making and improving compliance with EU legislation.

Useful data analysis is only feasible when inspection results are standardised. There are different ways of standardising inspection results. A standardised inspection form, including different categories which summarise all of the legal requirements, can be used during inspection. About 60% of the authorities included in our study use standard forms during inspection and 11% of them use a scoring system. Quantification of inspection results has been a matter of debate [20]. Studies have shown that the public interprets the grading inspection results [21] in a variety of ways and that there is a need for the improvement of consumer understanding of the inspection scores and the limitations of regulatory inspections [22]. It has been concluded that if a scoring system is implemented, it should be representative, easily computed and understood by operators and consumers. It should be amenable to statistical analysis and give a benchmark from which operators are able to improve legal compliance and authorities are able to improve the inspection systems [23]. If inspection results are published, they need to be understood by the public, taking into consideration that minimal information limited to a numerical score or grade may be misinterpreted [21]. A carefully designed, standardised inspection form and risk-based grading system for ships acceptable by all the EU competent authorities would ensure: a) consistent application of inspection procedures, b) avoiding of subjective application of standards, c) recording of the inspection findings in a consistent manner and d) data analysis and opportunities for system improvement.

The majority of the EU competent authorities that responded to our study believe that training, especially on ship hygiene issues, is needed for the public health personnel in ports. According to our study, few authorities provide training to inspectors on hygiene issues and only few authorities undertake ship specific training. Training is also an essential tool for standardising inspection procedures. It is normally provided to new inspectors before performing inspections alone, while formal periodic refresher training after the initial training should be available for all inspectors, regardless of length of experience [19]. On the other hand, training of crew members' depending on their work activities is also important. A study conducted aboard 22 ferries has revealed a clear improvement of the food safety practices following a refresher course and a worsening of practices after a crew change [24]. Other studies conducted in land-based premises have shown that food businesses that ensure food handlers have received training have improved their inspection scores [25]. EU legislation requires training for food handlers commensurate to their tasks [1]. The currently implemented EU SHIPSAN TRAINET project will produce training material for crew members and for public health professionals working in port health http://www.shipsan.eu.

The communication of inspection results between ports is essential, especially if an outbreak occurs on board a ship. A central database, where competent authorities can record and access inspection results, would provide a useful tool for data sharing. This would help to implement a common inspection plan throughout the EU and avoid the problems associated with under inspections or repetition of inspections.

## Conclusion

Our study has explored hygiene inspection practices on passenger ships in the EU. The study results have revealed that there are diverse approaches and practices related to inspection as well as legislation applied during inspections among the EU countries. It has revealed useful information for the public health authorities, the industry and decision and policy makers. The findings of this study are useful for developing a common integrated European inspection system in order to: a) minimise the risk of transmission of communicable diseases, b) improve levels of hygiene on board passenger ships as well as the level of compliance with existing EU legislation, c) ensure the provision of safe water and food, environment, air conditioning and other services to consumers and d) demonstrate the member countries' compliance with the European legislation. The EU SHIPSAN TRAINET project will put into action a pilot communication network among port health authorities, which will facilitate the exchange of information and will contribute to a coordinated response system in terms of ship-related health threats within the EU. It will further produce a manual including hygiene standards based on European legislation and will deliver training for hygiene issues to port health officers and crew members.

## Competing interests

Mel Skipp works for the Carnival cruise line. All other authors declare that they have no conflicts of interest.

## Authors' contributions

VM participated in the design of the study, data collection, analysis, interpretation of the results and drafted the manuscript. SW, GN, TR, MS and CLRB participated in the design of the questionnaire, data collection, results interpretation and editing of manuscript. JK participated in the design and coordination of the study and results interpretation. CH conceived of the study, participated in its design and coordination and results analysis and interpretation.

RM and CVS participated in the design of the study and supervised the study. EK and NB participated in the data collection and analysis. ISA, TM, GR, PM, CVM, CS, OS, CS, NP, JR, JA, NM, AK, VK, HCM, GS, MB, TP, GG, MDM, AS, DM and LH participated in the design of the questionnaire and data collection. All authors read and approved the final manuscript.

## Pre-publication history

The pre-publication history for this paper can be accessed here:

http://www.biomedcentral.com/1471-2458/10/122/prepub

## Supplementary Material

Additional file 1 **Questionnaire**. Questionnaire used for data collection of inspection practices Click here for file
