# Supplementary material for: Hygiene inspections on passenger ships in Europe - an overview
Source: BMC Public Health. 2010 Mar 10;10:122. doi: 10.1186/1471-2458-10-122 (PMC2847977; doi:10.1186/1471-2458-10-122)
Supplement: Additional file 1 — Questionnaire. Questionnaire used for data collection of inspection practices [file 1471-2458-10-122-S1.DOC]

### PROJECT TITLE: Assessing the usefulness of an EU ship sanitation programme and coordinated action for the control of communicable diseases in cruise ships and ferries.

### Work Package Description: 6: Collection, Analysis and Presentation of Data

### Work Package Leader: University of Thessaly, Greece

**General Guidelines for the completion of QUESTIONNAIRE B** – *“***Sanitation Inspections on board cruise ships and ferries amongst the EU countries”**

- **Questionnaire B** is accompanied by **Annex B**.
- Please tick the correct answer either in the questionnaire or in the Annex as requested.
- The annex is to be used to fill in details regarding **sanitation inspections** in your country**.** Please circle every time which question you are answering and describe synoptically in 2-3 lines the legislation or regulation or guideline stating the title, entry into force and amendment dates. Also please give 3-4 keywords or phrases related to the legislation, regulation or guideline. Finally, please send us an **electronic format of the documents referred to the Annex**. If there is not an electronic format available, please provide the original documents.
- The word **“cruise ship”** indicates a vessel (sea/ river/ lake-going) which carries more than twelve passengers who travel for pleasure.
- The word **“ferry”** indicates a vessel (sea/ river/ lake-going) which carries more than twelve passengers and is designed to move people and, often, vehicles on regular itineraries from one place to another.
- The word **“port”** means a seaport or a port on an inland body of water where ships arrive or depart.
- To assist you with the completion of the questionnaire **specific guidelines** for every question have been attached at the end of the questionnaire. Before answering the questions please read the specific guidelines!

PLEASE SEND YOUR COMPLETED QUESTIONNAIRES TO:

**Ass. Prof. Christos Hadjichristodoulou,** SHIPSAN Scientific Coordinator **Email:** xhatzi@med.uth.gr

For further assistance please contact:

**Ass. Prof. Christos Hadjichristodoulou**

SHIPSAN Scientific Coordinator

**Address:** SHIPSAN Project, University of Thessaly, Lapithon 6, Larissa, 41221, Greece

**Tel:** 0030 2410 565259 **Fax:** 0030 2410 258197 **Email:** xhatzi@med.uth.gr

| ***QUESTIONNAIRE (B)* - SANITATION INSPECTIONS ON BOARD CRUISE SHIPS AND FERRIES AMONGST THE EU COUNTRIES** |
| --- |

### PURPOSE OF QUESTIONNAIRE: Collection of information on sanitation inspections on board cruise ships and ferries among the EU countries - for assessing the usefulness of an EU strategy.

**PLEASE READ THE SPECIFIC GUIDANCE IN ANNEX B BEFORE COMPLETING THIS QUESTIONNAIRE**

| **RESPONDING AUTHORITY** |
| --- |
|  National central authority  Regional or Local Authority |
| 1. Name of the authority: 2. Telephone of the authority: 3. Address of the authority: 4. E-mail of the authority: 5. Contact person: |

1. Which aspect(s) do you have the **power to inspect** on board cruise ships and/or ferries?

Food safety

Potable water safety

Recreational water safety

Medical facilities

Accommodation spaces

Waste management

Pest Control

Housekeeping

Air handling and ventilation

Other Please specify:…………………. …………

1. What national legislation or regulation or guidelines related to **sanitation inspections on hygiene issues** are currently used on board of cruise ships and/or ferries?

□ Specific legislation for cruise ships and ferries (PLEASE FILL IN THE DETAILS IN ANNEX B)

□ National legislation or regulations of guidelines for land based premises including specific provisions for cruise ships and ferries. (PLEASE FILL IN THE DETAILS IN ANNEX B)

□ National legislation or regulations of guidelines for land based premises also applicable to cruise ships and ferries

□ Other (Please specify)……………………………………… (PLEASE FILL IN THE DETAILS IN ANNEX B)

1. What national legislation or regulation or guidelines related to **obtaining a permit** for food premises (restaurants, bars) are currently used that can be applied on board of cruise ships and/or ferries?

□ Specific legislation for cruise ships and ferries (PLEASE FILL IN THE DETAILS IN ANNEX B)

□ National legislation or regulations or guidelines for land based premises including specific provisions for cruise ships and ferries. (PLEASE FILL IN THE DETAILS IN ANNEX B)

□ National legislation or regulations or guidelines for land base premises also applicable to cruise ships and ferries

□No Legislation exists

□ Other (Please specify)………………………………………(PLEASE FILL IN THE DETAILS IN ANNEX B)

1. If **Yes**, how many cruise ships and/or ferries have applied for a permit in 2006?

Number of applicants in 2006: ……………………………………..…………………………………………………..

1. Inspection on board cruise ships and/or ferries are conducted on **international** voyages:

When asked to issue a certificate

During outbreak investigation

When investigating complains for unsanitary conditions

According to a specific routine program. Please specify:………………………………………………………………..

Other. Please specify:………………………………………………………………………………………………………..

1. Inspection on board cruise ships and/or ferries are conducted on a **national** voyage:

When asked to issue a certificate

During outbreak investigation

When investigating complains for unsanitary conditions

According to a specific routine program. Please specify:……………………………………..

Other. Please specify:……………………………………..

1. When you **board** a ship on an **international** voyage to carry out inspections, which laws do you have the power to enforce (except for the IHR)?

 Specific national legislation for ships

 National legislation for land based establishments

 There is no legislation to enforce aboard ships on international voyage during inspections

 Other. Please specify: …………………………………………………………

1. When you **board** a ship on a **national** voyage to carry out inspections, which laws do you have the power to enforce?

Specific national legislation for ships

National legislation for land based establishments

There is no legislation which I can enforce to ships on national voyage

Other. Please specify: ……………………………………………………………………………….……………………

1. Do you conduct **environmental sampling** (potable, recreational water **etc**) on board cruise ships and/or ferries?

Yes No

If Yes, please specify:

According to a specific routine program

During an outbreak investigation or when investigating complains

Other. Please specify: …………………………………………………………………………………………..…………

…………………………………………………………………………………………..…………

1. Do you conduct onboard measurements or manual tests (for example food temperature, water chlorination, etc) during inspections on cruise ships and/or ferries?

Yes No If Yes, please specify: ……………………………………………………………………………………

1. When the inspection or the environmental sampling results are unsatisfactory, is there any **port-to-port communication** to inform about the results the next port of call of the ship?

Yes, there is a port-to-port communication among the **national** port authorities

No, there is not a port-to-port communication among the **national** port authorities

Yes, there is a port-to-port communication with port authorities of other countries

No, there is not a port-to-port communication with port authorities of other countries

Other. Please specify: …………………………………………………………………………

1. Do you use **standardised inspection forms** (checklists) during the inspections?

□ **YES** (PLEASE SPECIFY BELOW AND ATTACH A COPY) □ **NO** (CONTINUE TO QUESTION B13)

SPECIFY THE CATEGORIES THE INSPECTION CHECKLIST COVERS: ……………………………………………………………………………

……………………………………………………………………………………………………………………………………………………………………

SPECIFY THE NUMBER OF ITEMS INCLUDED: ……………………………………………………………………………………………………….

…………………………………………………………………………………………………………………………………………………………………

1. Is the **sanitation inspection** on hygiene issues **scored**?

□**YES** (PLEASE SPECIFY BELOW) □**NO** (CONTINUE TO QUESTION B14)

SPECIFY THE SUCCESS SCORE: ……………………………………………………………………………...…

SPECIFY THE FAILURE SCORE: ………………………………………………………………………………….

1. Are the **inspection results** collected and centrally analysed?

□ **YES** (PLEASE SPECIFY BELOW THE AUTHORITY) □ **NO** (CONTINUE TO QUESTION B15)

Name of the authority: ………………………………………………………………………………………

Telephone of the authority: …………………………………………………………………………………..

1. Are the **inspection results** publicised (web etc.)?

□ **YES** (PLEASE SPECIFY BELOW) □ **NO** (CONTINUE TO QUESTION B16)

………………………………………………………………………………………………

………………………………………………………………………………………………

1. How many inspections did you carry out on board cruise ships and/or ferries during 2006?

**Number of Inspections during 2006** …………………………………………….……………...

1. Are there any specific sanitation issues considered **critical** enough to issue an **order that a ship does not sail?**

□ **YES** (PLEASE SPECIFY BELOW) □ **NO** (CONTINUE TO QUESTION B18)

………………………………………………………………………………………………………………………………

………………………………………………………………………………………………………………………………

………………………………………………………………………………………………………………………………

1. Were public health professionals responsible for sanitation inspections on cruise ships and/or ferries in port authorities of your country **trained on sanitation inspections during the last three years**?

□ **YES** (PLEASE SPECIFY BELOW AND CONTINUE TO B19)□ **NO** (CONTINUE TO QUESTION B19)

SPECIFY MAIN TOPICS OF TRAINING ……………………………………………………………………………………………………………………..

……………………………………………………………………………………………………………………..

1. Do the health professionals in port authorities of your country undertake **specific** **training on ship sanitation inspections** on board cruise ships and/or ferries?

□ **YES** (PLEASE SPECIFY BELOW AND CONTINUE TO B20) □ **NO** (CONTINUE TO QUESTION B20)

SPECIFY MAIN TOPICS OF TRAINING ……………………………………………………………………………..

……………………………………………………………………………………………………………………..

……………………………………………………………………………………………………………………..

1. If your answer to the previous question is **No,** do you think that specific training on ship sanitation is needed?

□ **YES** (PLEASE SPECIFY BELOW AND CONTINUE TO B21) □ **NO** (CONTINUE TO QUESTION B21)

PLEASE SPECIFY what do you think the training should include

………………………………………………………………………………………………………………………………

………………………………………………………………………………………………………………………………

………………………………………………………………………………………………………………………………

1. In your opinion are there any **gaps in sanitation inspections on hygiene issues** on board cruise ships and/or ferries?

□ **YES** (Please specify the gaps and outline your suggestions) □ **NO**

……………………………………………………………………………………………………………………

……………………………………………………………………………………………………………………

……………………………………………………………………………………..…………………………….

…………………………………………………………………………………..………………………………

**ANNEX B**

PLEASE DESCRIBE SYNOPTICALLY IN 2-3 LINES ANY SPECIFIC LEGISLATION AND / OR GUIDELINES STATING THE TITLE, DATE, AMENDMENT DATE AND GIVE 3-4 KEYWORDS OR PHRASES. PROVIDE THE ELECTRONIC FORMAT OF THE DOCUMENTS. If there is not an electronic format available, please provide the original documents. (Please send us the complete legislation document incorporating all the amendments. Otherwise send the original law document attaching all the standing amendments).

**QUESTION B2 B3** (PLEASE CIRCLE WHICH QUESTION YOU ARE ANSWERING)

TITLE / MAIN CONCEPT: …………………………………………………………………………………………..….….…..

………………………………………………………………………………………...…………………………………….

………………………………………………………………………………………………………………………………

ENTRY INTO FORCE: ..........................................MODIFICATION/ AMENDMENT DATES: ................................................................

IF **GUIDELINES** PLEASE SPECIFY: □ MANDATORY □ SCIENTIFIC

KEY WORDS OR PHRASES: …………………………………………………………………………………………………………….

………………………………………………………………………….………………………………………………………………………………………………….

..………………………………………………………………………………………………………………………….………………………………………………..

**Does this legislation/guideline apply to:** □ Ships under National flag □ Ships under Non National flag

□Ship with national itinerary □Ships with international itinerary

**QUESTION B2 B3** (PLEASE CIRCLE WHICH QUESTION YOU ARE ANSWERING)

TITLE / MAIN CONCEPT: …………………………………………………………………………………………..….….…..

………………………………………………………………………………………...…………………………………….

………………………………………………………………………………………………………………………………

ENTRY INTO FORCE: ..........................................MODIFICATION/ AMENDMENT DATES: ................................................................

IF **GUIDELINES** PLEASE SPECIFY: □ MANDATORY □ SCIENTIFIC

KEY WORDS OR PHRASES: …………………………………………………………………………………………………………….

………………………………………………………………………….………………………………………………………………………………………………….

..………………………………………………………………………………………………………………………….………………………………………………..

**Does this legislation/guideline apply to:** □ Ships under National flag □ Ships under Non National flag

□Ship with national itinerary □Ships with international itinerary

**QUESTION B2 B3** (PLEASE CIRCLE WHICH QUESTION YOU ARE ANSWERING)

TITLE / MAIN CONCEPT: …………………………………………………………………………………………..….….…..

………………………………………………………………………………………...…………………………………….

………………………………………………………………………………………………………………………………

ENTRY INTO FORCE: ..........................................MODIFICATION/ AMENDMENT DATES: ................................................................

IF **GUIDELINES** PLEASE SPECIFY: □ MANDATORY □ SCIENTIFIC

KEY WORDS OR PHRASES: …………………………………………………………………………………………………………….

………………………………………………………………………….………………………………………………………………………………………………….

………………………………………………………………………….………………………………………………………………………………………………….

**Does this legislation/guideline apply to:** □ Ships under National flag □ Ships under Non National flag

□Ship with national itinerary □Ships with international itinerary

**SPECIFIC GUIDELINES FOR**

**QUESTIONNAIRE B** – *“***Sanitation Inspections on board cruise ships and ferries amongst the EU countries**

1. Please tick every answer which is applicable to your situation. In the case that you do not know or that you are not responsible please state it in the “Other” section.
2. In Annex B provide details (e.g. title, entry into force etc.) of any national legislation or regulations.

**“Sanitation inspections”** indicates a formal or official examination which is conducted by officers of a competent authority on board cruise ships and ferries regarding food premises permit, food safety, drinking water safety and recreational water safety (including food and water quality monitoring), food handlers’ hygiene, pest control and housekeeping to determine compliance with legislation.

1. In Annex B provide details (e.g. title, entry into force etc.) of any national legislation or regulations.

A “**permit for food premises (restaurants, bars)”** on board of cruise ships and ferries is an official document issued after examination of compliance with sanitation standards during all phases of food handling and storage.

1. Please specify the **number of permits** for food premises on board of cruise ships and ferries you have issued.
2. Please tick every answer which is applicable to your situation. In the case that you do not know or that you are not responsible please state it in the “Other” section.

**“International Voyage”** means a voyage by sea from a port of a Member State to a port outside that Member State, or conversely.

1. Please tick every answer which is applicable to your situation. In the case that you do not know or that you are not responsible please state it in the “Other” section.

**“National Voyage”** means a voyage in sea areas from a port of a Member State to the same or another port within that Member State.

1. Please tick every answer which is applicable to your situation. In the case that you do not know or that you are not responsible please state it in the “Other” section.

By **“laws”** we mean national legislation or regulation or guidelines.

1. Please tick every answer which is applicable to your situation. In the case that you do not know or that you are not responsible please state it in the “Other” section.

By **“laws”** we mean national legislation or regulation or guidelines

1. Please tick every answer which is applicable to your situation. In the case that you do not know or that you are not responsible please state it in the “Other” section.

**“Recreational water”** means water from swimming pools, spas, hot tubs, decorative water fountains, lakes, rivers, or oceans.

**"Potable water"** means: (a) all water **intended for human consumption** either in its original state or after treatment, intended for drinking, cooking, food preparation or other domestic purposes, regardless of its origin and whether it is supplied from a distribution network, from a tanker, or in bottles or containers; (b) all water used in any food-production undertaking for the manufacture, processing, preservation or marketing of products or substances intended for human consumption unless the competent national authorities are satisfied that the quality of the water cannot affect the wholesomeness of the foodstuff in its finished form (DIRECTIVE 98/83/EC).

**“Environmental sampling” means** the process of collecting representative samples of material from the natural environment, such as, potable water, recreational water, food, air. Such samples are subsequently analyzed in order to determine their composition or contamination.

1. Please specify whether you conduct onboard measurements or manual tests (for example of food temperature using thermometers, or free residual chlorine using test kits or other appropriate devices etc) during inspections on cruise ships and ferries.
2. Please tick every answer which is applicable to your situation regarding the communication procedure you follow when the environmental sampling (e.g. sampling recreational or potable water) you conduct provides unsatisfactory results. In the case that you do not know or that you are not responsible please state it in the “Other” section.
3. Please specify the numbers of items included in the standardized inspection form (checklist) and the categories it covers. Please attach a copy of the standardized inspection form.

The word “**item**” means the part of the standardized inspection report which describes the specific point of the inspection.

The word “categories” means the topic which includes the individual items in the inspection report. For example, the “Food safety” category might include the item concerning food temperature control, the item concerning food handlers infection etc.

1. Please provide information on whether the standardized inspection form (checklist) used for the sanitation inspections includes a **scoring system** and what is the scale of it.
2. Please answer whether the data collected from the inspections are analysed by an authority and provide their details.

The word **“authority”** indicates any independent service or department within a government ministry.

1. If the results of the inspections are publicized either in a journal or the internet please specify where.
2. Please specify the number of inspections carried out on board cruise ships and ferries during 2006.
3. Please list the critical items of the sanitation inspection aboard cruise ships or ferries that if they were to be found unsatisfactory would lead to an **order for not sailing**.
4. If the public health professionals in port authorities of your country undertook training **(courses, seminars, etc)** related to sanitation inspections **generally**, the last two years please outline the main topics covered by the training. **For example**: food safety, drinking water safety and recreational water safety (including food and water quality monitoring), food handlers’ hygiene, pest control and housekeeping.
5. If the public health professionals in port authorities of your country undertook training **(courses, seminars, etc)** related to sanitation inspections **specifically** for ships, please outline the main topics covered by the training. **For example**: food safety, drinking water safety and recreational water safety (including food and water quality monitoring), food handlers’ hygiene, pest control and housekeeping.
6. If the professionals in port authorities of your country **do not** undertake training related to sanitation inspections on board cruise ships and ferries please indicate if you think that specific training for ships is needed and outline the subject areas you think they should be trained on. **For example**: food safety, drinking water safety and recreational water safety (including food and water quality monitoring), food handlers’ hygiene, pest control and housekeeping.
7. **“Gaps”** means any point that should be legislated, but it is not covered by EU or national legislation or when the legislation does not specify its applicability to cruise ships and ferries.
